# PRC1 and RACGAP1 are Diagnostic Biomarkers of Early HCC and PRC1 Drives Self-Renewal of Liver Cancer Stem Cells

**DOI:** 10.3389/fcell.2022.864051

**Published:** 2022-04-04

**Authors:** Shixin Liao, Kaili Wang, Lulu Zhang, Gaoli Shi, Zhiwei Wang, Zhenzhen Chen, Pingping Zhu, Qiankun He

**Affiliations:** School of Life Sciences, Zhengzhou University, Zhengzhou, China

**Keywords:** liver cancer, cancer stem cell, self-renewal, metastasis, biomarker

## Abstract

Hepatocellular carcinoma (HCC) is the fourth leading cause of cancer-related deaths across the world. Due to the lack of reliable markers for early HCC detection, most HCC patients are diagnosed in middle/late stages. Liver cancer stem cells (CSCs), which are drivers of liver tumorigenesis, usually emerge in the early HCC stage and are also termed as liver tumor initiation cells (TIC). Liver CSCs contribute to initiation, propagation, and metastasis of HCC and also play a key role in tumor therapy. Taking advantage of online-available data sets, bioinformatic analyses, and experimental confirmation, here we have screened out PRC1 and RACGAP1 as reliable markers for early HCC detection. PRC1 or RACGAP1 knockdown dramatically inhibited the proliferation, migration, and invasion capacities of HCC cells, conferring PRC1 and RACGAP1 as predominant modulators for HCC propagation and metastasis. Moreover, the sphere formation capacity of HCC cells was impaired after PRC1 knockdown, revealing the function of PRC1 as a modulator for liver CSC self-renewal. Furthermore, the inhibitor of PRC1 had same phenotypes as PRC1 knockdown in HCC cells. Altogether, PRC1 and RACGAP1 are identified both as prognosis markers for early HCC detection and therapeutic targets for liver cancer and liver CSCs, adding additional layers for the early prognosis and therapy of HCC.

## Introduction

In China, the incidence of liver cancer has doubled in the past two decades. However, the HCC which accounts for 85–90% of liver cancer is still difficult to detect in the early stage using conventional tumor diagnosis methods, such as computed tomography (CT), magnetic resonance imaging (MRI), and ultrasonic inspection (Roberts et al., 2018; Xie et al., 2020). The HCC patients are already in the advanced HCC stage when they are diagnosed, which largely increases the difficulty of tumor therapy and the risk of tumor-related death. In addition, due to the lack of effective means to detect the therapeutic effect, the tumor will relapse and metastasize after treatment, resulting in the death of the patient (Wu et al., 2021). Therefore, it is urgent to explore more effective methods for early diagnosis and prognostic detection of HCC.

Recently, accumulating biomarkers have been identified for tumor diagnosis and prognosis. Some tumor-specific genes are expressed in very early stages in tumorigenesis, whereas others are expressed in later stages. In addition to gene expression, gene mutation, copy number, DNA methylation, and protein levels have also been reported as biomarkers for tumor diagnosis and prognosis (Cheng et al., 2018). However, many biomarkers are obtained from limited tumor samples and thus cannot represent comprehensive features of HCC samples. With the rapid development of computer science and the rapid accumulation of biological data, bioinformatics has emerged as an efficient tool for the analysis of huge amounts of biological data. Microarray and RNA sequencing are predominant strategies in the rapid development of bioinformatics and could be used for a variety of genomics and proteomics analyses (Gao et al., 2019; Chen et al., 2020). At present, a large number of microarray data sets have been deposited in several databases, but valuable information needs to be analyzed by bioinformatics. In our study, three cohorts, namely, GSE102079, GSE112790, and GSE121248, were selected from the GEO data set for the DEG analysis as testing cohorts, and other four cohorts (GSE45267, GSE62232, GSE84402, and GSE6764) were used to confirm our results. Taking advantage of GO, KEGG, PPI, and LASSO, we finally identified PRC1, RACGAP1, CENPF, and CCNB2 as diagnosis markers of early HCC detection.

In addition to the lake of biomarkers, the significant heterogeneity of HCC also accounts for the poor prognosis of HCC patients (Zhang et al., 2019). The hierarchical organization of tumor cells within the tumor bulk is largely generated from a small subset of cells termed cancer stem cells (CSCs) (Batlle and Clevers, 2017). Liver CSCs usually emerge in the very early stage of HCC and contain several subsets (Zheng et al., 2018). With the ability of self-renewal and differentiation, liver CSCs contribute to tumor initiation, metastasis, drug resistance, and tumor relapse (Nio et al., 2017; Lee et al., 2022). Liver CSCs can survive in various clinical therapies, including chemotherapy, radiotherapy, and up-to-date immunotherapy, and differentiate into new tumors (Zheng et al., 2018). The self-renewal of liver TICs is regulated by several signaling pathways, including Wnt/β-catenin (Chen et al., 2018a), Notch (Zhu et al., 2015a), Hedgehog (Wu et al., 2019), Hippo/Yap (Guo et al., 2017), and PKC (Chen et al., 2018b) signaling pathways, which are further modulated by various intracellular and niche factors. Recently, we have identified several functional liver CSC-intrinsic factors, including C8orf4 (Zhu et al., 2015a), ZIC2 (Zhu et al., 2015b), lnc-β-Catm (Zhu et al., 2016), and cia-MAF (Chen et al., 2021). We have also proved that non-CSC secreted WNT5A drives the activation of Wnt/β-catenin signaling in liver CSCs (Chen et al., 2018a). However, the previous studies in the CSC field largely depend on a few clinical samples and many discoveries are only suitable for some patients but not for others, largely limiting the clinical applications for highly heterogenous HCC patients. Combining unbiased genome-wide screening with a large number of HCC samples and CSC detection, we have identified PRC1 and RACGAP1 as predominant modulators for HCC propagation and metastasis.

## Materials and Methods

### Acquisition of Data Resources

We entered the keyword “HCC” in GEO data sets of NCBI (https://www.ncbi.nlm.nih.gov/geo/) for retrieval and got 20603 results. “Expression Profiling by Array” in the study type and “Homo Sapiens” in top organisms were added to the filters. Finally, 417 results were obtained. We selected sequencing data sets of HCC and a normal liver with a sample size greater than 100 on the same platform. Then three gene expression profiles [GSE102079 (Chiyonobu et al., 2018), GSE112790 (Shimada et al., 2019), and GSE121248 (Wang et al., 2007)] were selected. These three microarrays were based on GPL570 (HG-U133_Plus_2). The GSE102079 cohort consisted of 152 HCC samples and 91 non-cancer samples from 152 patients. The GSE112790 cohort consisted of 183 HCC samples and 15 non-cancer samples from 183 patients. The GSE121248 included 70 HCC samples and 30 normal samples from 107 patients. The basic features of GSE102079 and GSE112790 data sets have not been disclosed. The basic features of GSE121248 data sets are summarized in Table 1.

**TABLE 1:** Basic features of the GSE121248 data set.

GEO accession	Tissue	Sex	Age	HBV status
GSM3428716	Tumor sample	M	72	Positive
GSM3428717	Tumor sample	M	39	Positive
GSM3428718	Tumor sample	M	63	Positive
GSM3428719	Tumor sample	M	44	Positive
GSM3428720	Tumor sample	M	43	Positive
GSM3428721	Tumor sample	M	38	Positive
GSM3428722	Tumor sample	M	47	Positive
GSM3428723	Tumor sample	F	74	Positive
GSM3428724	Tumor sample	M	75	Positive
GSM3428725	Tumor sample	F	58	Positive
GSM3428726	Tumor sample	M	55	Positive
GSM3428727	Tumor sample	M	51	Positive
GSM3428728	Tumor sample	M	51	Positive
GSM3428729	Tumor sample	M	67	Positive
GSM3428730	Tumor sample	M	66	Positive
GSM3428731	Tumor sample	M	67	Positive
GSM3428732	Tumor sample	M	76	Positive
GSM3428733	Tumor sample	M	50	Positive
GSM3428734	Tumor sample	M	30	Positive
GSM3428735	Tumor sample	M	54	Positive
GSM3428736	Tumor sample	M	33	Positive
GSM3428737	Tumor sample	M	61	Positive
GSM3428738	Tumor sample	M	51	Positive
GSM3428739	Tumor sample	M	38	Positive
GSM3428740	Tumor sample	M	79	Positive
GSM3428741	Tumor sample	M	68	Positive
GSM3428742	Tumor sample	M	59	Positive
GSM3428743	Tumor sample	M	72	Positive
GSM3428744	Tumor sample	M	57	Positive
GSM3428745	Tumor sample	F	52	Positive
GSM3428746	Tumor sample	M	57	Positive
GSM3428747	Tumor sample	F	35	Positive
GSM3428748	Tumor sample	M	57	Positive
GSM3428749	Tumor sample	M	33	Positive
GSM3428750	Tumor sample	F	60	Positive
GSM3428751	Tumor sample	M	69	Positive
GSM3428752	Tumor sample	M	48	Positive
GSM3428753	Tumor sample	M	75	Positive
GSM3428754	Tumor sample	M	66	Positive
GSM3428755	Tumor sample	M	59	Positive
GSM3428756	Tumor sample	M	44	Positive
GSM3428757	Tumor sample	M	66	Positive
GSM3428758	Tumor sample	M	83	Positive
GSM3428759	Tumor sample	M	64	Positive
GSM3428760	Tumor sample	M	62	Positive
GSM3428761	Tumor sample	M	73	Positive
GSM3428762	Tumor sample	F	72	Positive
GSM3428763	Tumor sample	M	39	Positive
GSM3428764	Tumor sample	M	69	Positive
GSM3428765	Tumor sample	M	35	Positive
GSM3428766	Tumor sample	M	62	Positive
GSM3428767	Tumor sample	M	59	Positive
GSM3428768	Tumor sample	M	64	Positive
GSM3428769	Tumor sample	M	70	Positive
GSM3428770	Tumor sample	M	62	Positive
GSM3428771	Tumor sample	M	56	Positive
GSM3428772	Tumor sample	M	81	Positive
GSM3428773	Tumor sample	M	77	Positive
GSM3428774	Tumor sample	M	72	Positive
GSM3428775	Tumor sample	F	74	Positive
GSM3428776	Tumor sample	F	72	Positive
GSM3428777	Tumor sample	M	64	Positive
GSM3428778	Tumor sample	M	75	Positive
GSM3428779	Tumor sample	M	51	Positive
GSM3428780	Tumor sample	M	56	Positive
GSM3428781	Tumor sample	M	80	Positive
GSM3428782	Tumor sample	M	55	Positive
GSM3428783	Tumor sample	M	52	Positive
GSM3428784	Tumor sample	F	76	Positive
GSM3428785	Tumor sample	M	53	Positive
GSM3428786	Adjacent normal sample	F	58	Positive
GSM3428787	Adjacent normal sample	M	51	Positive
GSM3428788	Adjacent normal sample	M	67	Positive
GSM3428789	Adjacent normal sample	M	66	Positive
GSM3428790	Adjacent normal sample	M	67	Positive
GSM3428791	Adjacent normal sample	M	76	Positive
GSM3428792	Adjacent normal sample	M	50	Positive
GSM3428793	Adjacent normal sample	M	30	Positive
GSM3428794	Adjacent normal sample	M	54	Positive
GSM3428795	Adjacent normal sample	M	33	Positive
GSM3428796	Adjacent normal sample	M	61	Positive
GSM3428797	Adjacent normal sample	M	51	Positive
GSM3428798	Adjacent normal sample	M	38	Positive
GSM3428799	Adjacent normal sample	M	79	Positive
GSM3428800	Adjacent normal sample	M	68	Positive
GSM3428801	Adjacent normal sample	M	59	Positive
GSM3428802	Adjacent normal sample	M	72	Positive
GSM3428803	Adjacent normal sample	M	57	Positive
GSM3428804	Adjacent normal sample	F	52	Positive
GSM3428805	Adjacent normal sample	M	57	Positive
GSM3428806	Adjacent normal sample	F	35	Positive
GSM3428807	Adjacent normal sample	M	57	Positive
GSM3428808	Adjacent normal sample	M	62	Positive
GSM3428809	Adjacent normal sample	M	56	Positive
GSM3428810	Adjacent normal sample	M	81	Positive
GSM3428811	Adjacent normal sample	M	77	Positive
GSM3428812	Adjacent normal sample	M	72	Positive
GSM3428813	Adjacent normal sample	F	74	Positive
GSM3428814	Adjacent normal sample	F	72	Positive
GSM3428815	Adjacent normal sample	M	64	Positive
GSM3428816	Adjacent normal sample	M	75	Positive
GSM3428817	Adjacent normal sample	M	51	Positive
GSM3428818	Adjacent normal sample	M	56	Positive
GSM3428819	Adjacent normal sample	M	80	Positive
GSM3428820	Adjacent normal sample	M	55	Positive
GSM3428821	Adjacent normal sample	M	52	Positive
GSM3428822	Adjacent normal sample	F	76	Positive

### Data Analysis

GEO2R (https://www.ncbi.nlm.nih.gov/geo/geo2r/) was used for the DEG analysis. We typed the number of the selected gene microarrays in GEO data sets of NCBI and clicked “analyze with GEO2R” to select HCC samples and non-cancer samples for analysis. The *p* value < 0.05 and |logFC| > 1.5 were used as the selection criteria.

### Functional Annotation of DEGs

The GO analysis provides gene characters about cellular components (CCs), molecular functions (MFs), and biological processes (BPs). KEGG, a well-known database containing many signaling pathways, and it links the list of genes to the enrichment of signaling pathways. The DEGs could be analyzed by GO and KEGG on DAVID (https://david.ncifcrf.gov/) (Huang et al., 2009a; Huang et al., 2009b). *p* < 0.05 was considered to be statistically significant.

### PPI Network Construction and hub Gene Identification

PPI evaluates the functional interactions between proteins through their interaction networks. The Matthews correlation coefficient (MCC), a parameter indicating correlation, can be used to screen for genes with high correlation. The Search Tool for the Retrieval of Interacting Genes (STRING) (http://string-db.org/) (Szklarczyk et al., 2021) is an online database for analysis and visualization of protein–protein interactions. We downloaded the PPI list from STRING and imported it to Cytoscape (www.cytoscape.org/); (Shannon et al., 2003) the proteins with higher MCC tend to play a more important role. cytoHubba, which could calculate MCC of each protein, is a plug-in of Cytoscape. After calculation with cytoHubba, we selected the top 30 proteins with the highest MCC as hub proteins in this study.

### LASSO

We used R version 4.0.3 to complete the lasso regression analysis, and the R package used is glmnet. LASSO (Tibshirani, 2011) regression is characterized by variable selection and regularization while fitting the generalized linear model. The degree of LASSO regression complexity adjustment is controlled by the parameter λ. The larger the parameter λ, the greater the penalty for a linear model with more variables, resulting in a model with fewer variables.

### Confirmation With Validation Cohorts

Three gene expression profiles [GSE45267 (Chen et al., 2018c), GSE62232 (Schulze et al., 2015), and GSE84402 (Wang et al., 2017)] were selected from the GEO database as validation cohorts. Common DEGs and top 30 hub genes were obtained as described before. In addition, we analyzed gene expression in the normal liver, liver cirrhosis, and very early HCC tissues using the data set GSE6764 (Wurmbach et al., 2007).

### Gene Expression Profiling Interactive Analysis

The gene expression profiling interactive analysis (GEPIA) (http://gepia.cancer-pku.cn/) (Tang et al., 2017), an open interactive website, could perform a personalized analysis according to user’s need in expression results of RNA sequencing of 9736 tumor samples and 8587 normal samples in TCGA and GTEx databases. We performed a single gene analysis for all hub genes in 369 HCC samples and 160 normal liver samples. |log2FC| > 1; *p* < 0.05 was considered to be statistically significant.

### Kaplan–Meier Survival Analysis of hub Genes

The Kaplan–Meier plotter (http://kmplot.com/analysis/) (Nagy et al., 2018) is a website that contains a lot of clinical characteristics about patients with 21 types of cancer (breast cancer, lung cancer, liver cancer, etc.) and could be used to assess the impact of 54K genes on survival in these cancers. In our study, the Kaplan–Meier plotter was used to analyze liver cancer survival. The hazard ratio (HR) with 95% confidence interval (CI) and log rank *p* value were calculated and displayed on the plot. Median is used as the cutoff criterion for the survival analysis.

### DepMap

The DepMap (https://depmap.org/portal/) portal (Tsherniak et al., 2017; Dwane et al., 2021) empowers the research community to make discoveries related to cancer vulnerabilities by providing open access to key cancer dependencies using analytical and visualization tools. DepMap contains 796 CRISPR screening and 710 RNAi screening data sets, which can show the necessity of genes in various cells. By inputting PRC1, RACGAP1, CENPF, and CCNB2 into the DepMap database, the effect of knockout or knockdown genes on survival of different cell lines (including hepatoma cell lines) could be analyzed.

### Immunohistochemistry

The protein expression levels of PRC1 and RACGAP1 were detected by immunohistochemistry in paraffin-embedded sections of 5-μm-thick liver cancer tissue and adjacent normal tissue, as described (Chen et al., 2016). Paraffin-embedded sections were first taken for dewaxing and antigen repair, and then the samples were incubated with PRC1 or RACGAP1 antibodies (Proteintech, #15617-1-AP, #13739-1-AP) at 4°C overnight. After incubation, the slides were cleaned with phosphate-buffered saline with Tween 20 (PBST) and incubated with HRP-conjugated secondary antibodies (Proteintech, #SA00001-2) at room temperature for 1 h. After PBST cleaning, the slides were colored with DAB (ZSGB-BIO, #ZLI-9018). Then the slides were stained with hematoxylin staining solution (solarbio, #G1080), dehydrated, and sealed with a neutral gum. Finally, the slides were observed using the SLIDEVIEW VS200 slide scanner (Olympus).

### Cell Culture

Hep3B, HepG2, and HEK 293T were cultivated in Dulbecco’s Modified Eagle Medium (Gibco, #12800017) containing thermally inactivated fetal bovine serum (10%) (Lonsera, #S711-001S), penicillin (100 U/ml) (Sigma), and streptomycin (Sigma) (0.1 mg/ml) under a damp 5% CO_2_ atmosphere at 37°C.

### Gene Knockdown Vector Construction

We looked for targets on the GPP Web Portal (https://portals.broadinstitute.org/gpp/public) and designed shRNA with a fixed structure (target sequences: shPRC1-1: GCA​GGA​ACA​TTC​AAA​GGC​ATT​T; shPRC1-2: GCG​AGT​TAC​ATG​TTG​AGC​CAT; shRACGAP1-1: GCT​AGG​ACG​ACA​AGG​CAA​CTT​T; and shRACGAP1-2: GCA​GGT​GGA​TGT​AGA​GAT​CAA​A). shRNA primers were synthesized by BGI Tech Solutions (Beijing Liuhe). shRNA was linked to the enzyme-digestion pSicoR vector [Addgene, #11579]. The recombined plasmid was extracted using the endotoxin-free plasmid extraction kit and sequenced correctly.

### Lentiviral Infection

The lentiviral gene knockdown plasmid was co-transfected into HEK293T cells with lentiviral packaging plasmids psPAX2 (Addgene, #12260) and pMD2.G (Addgene, #12259). HEK293T cells were cultured in DMEM + 10% FBS and seeded in a 6-well cell culture plate (Corning, #3516) the day before transfection such that they would be 80–90% confluent at the time of transfection. One hour before transfection, the media was replaced with 2 ml of fresh prewarmed DMEM + 10% FBS. We added 20 μL of 2.5M CaCl2, 2 mg gene knockdown plasmid, 1.5 mg psPAX2, and 0.5 mg pMD2.G to 180 μL ddH2O. Then 200 μL HBS was added, mix thoroughly, and incubated at room temperature for 5 min. The mixture was then gently added to the 6-well cell culture plate. The transfection mix was aspirated after 8 h and replaced with fresh prewarmed DMEM + 10% FBS. Viral particles were harvested 48 h after the media was changed and filtered by a 0.22 μm syringe filter (Millipore, #SLGP033R). Liver cancer cells were cultured in DMEM + 10% FBS and seeded in a 6-well cell culture plate the day before transfection such that they would be 50% confluent at the time of transfection. The original medium was replaced with the filtered virus fluid; polybrene (Sigma) was added at 5 mg/ml and was replaced with fresh prewarmed DMEM + 10% FBS 6 h later.

### Quantitative RT-PCR

Total RNA was separated from HCC cell lines with an RNA isolater (Vazyme, #R401-01-AA), and its concentration was determined using NanoDrop ONE (Thermo Fisher Scientific). The RNA was then reverse-transcribed into cDNA using a reverse transcription kit (Vazyme, #R323-01). qRT-PCR detection was performed using the AceQ Universal SYBR qPCR Master Mix kit (Vazyme, #Q511-02) in the QuantStudio™ 5 Real-Time PCR (Thermo Fisher Scientific) (PRC1: forward primer: CGC​CAT​GAG​GAG​AAG​TGA​GG, reverse primer: TTG​TAA​CCG​CTG​GTC​CTC​TG; RACGAP1: forward primer: ACG​TTG​AAT​AGG​ATG​AGT​CAT​GGA, reverse primer: AAA​GTC​CTT​CGC​CAA​CTG​GA). All procedures were conducted in accordance with the manufacturer’s instructions. The thermal cycling parameters for amplification were as follows: pre-denaturation at 95°C for 10 min, denaturation at 95°C for 10 s, and annealing and extension at 60°C for 1 min for a total of 40 cycles.

### Western Blotting

Cells were lysed in protein lysate and were bathed in boiling water for 15 min. Proteins were resolved in a 10% polyacrylamide gel at 80 V for 120 min and transferred to a PVDF membrane (Millipore) at 18A for 60 min. The membrane was blocked for non-specific binding with 5% non-fat powdered milk in Tris-buffered saline with Tween 20 (TBST) for 1 h at room temperature and then probed with primary antibodies overnight at 4°C and with HRP-conjugated secondary antibodies for 1 h at room temperature. Blots were visualized with ECL detection (Meilunbio, # MA0186), and chemiluminescent signals were captured using the ChemiDoc™ MP Imaging System. Primary antibodies used were PRC1, RACGAP1, MYC-Tag (Cell Signaling Technology, #2276S), AXIN2 (Proteintech, #20540-1-AP), CCND1 (ABCAM, #AB16663), and MMP7 (Proteintech, # 3801S).

### Cell Proliferation

Hepatocarcinoma cells were isolated and seeded in 96-well plates (Corning, #353916) with 2000 cells per well. In the blank group, the medium containing FBS was added. The cells were cultured in a CO_2_ cell incubator (Thermo Fisher Scientific) for 24, 48, 72, and 96 h. Before testing, each well was changed into working solution (medium 100 μL, CCK-8 (Solarbio, #CA1210) solution 10 μL) under a dark condition. After incubation at 37°C for 1.5 h, the absorbance value at 450 nm (OD450) was measured with a microplate reader (Thermo Fisher Scientific, Multiskan FC).

### Flow Cytometry

We collected the cells and washed them with PBS. Then the cells were fixed with a fixation/permeabilization concentrate (Thermo Fisher Scientific, #00-5123-43) at 4°C for 0.5 h and permeated with a permeabilization buffer (Thermo Fisher Scientific, #00-8333-56) at 4°C for 5 min. Then the cells were incubated with Ki-67 APC direct-labeled antibody (Thermo Fisher Scientific, #17-5699-42) at room temperature for 20 min and washed with PBS. Flow cytometry was performed on the FACSymphony™S6 (BD Biosciences) platform, and results were analyzed using FlowJo software version 10.5.3.

### Immunofluorescence

Immunofluorescence was used to detect the expression level of Ki-67 in HCC cell slides to indicate cell proliferation ability. First, liver cancer cells were inoculated on the slides and fixed with 4% paraformaldehyde (Biosharp, #BL539A). The slides were then cleaned with PBS and incubated with the KI-67 APC direct-labeled antibody at room temperature for 1 h. After cleaning with PBS, the sample was sealed with an antifading mounting medium (with DAPI) (Solarbio, #S2110). Finally, the slides were observed using the SLIDEVIEW VS200 slide scanner (Olympus).

### Colony Formation Assay

Hepatocarcinoma cells were isolated and seeded in 6-well plates with 500 cells per well. After being cultured for 10 days, cell specimens in PBS were collected and fixed with 4% paraformaldehyde before being subjected to crystal violet staining (Solarbio, #G1062). The clone (>50 cells) number was measured using a scanner.

### Wound-Healing Assay

Confluent cells were scraped using a 10 μL pipette tip (Axygen, #AXYT300). After 24 h, the cells migrated to the wound and the scratched area were examined using an inverted microscope (Nikon, ECLIPSE Ti2). The migration rate was obtained by the formula: (Width0h−Width24h)/Width0h.

### Transwell

For the cell migration assay, the density of HCC cells was adjusted to 1 × 10^5^ cells/mL by using DMEM without FBS. We placed the Transwell permeable supports (Corning, #3422) into the 24-well plate (Corning, #3524), and added 600 μL DMEM containing 10% FBS to the lower chamber and 200 μL cell fluid to the upper chamber. After 24 h, the cells were fixed with 4% paraformaldehyde and stained with crystal violet. Finally, the cell was photographed using an inverted microscope.

For the cell invasion assay, serum-free DMEM was mixed with Matrigel (Corning, #354320) in 8:1 and evenly spread in the upper chamber of Transwell permeable supports. The chambers were then placed in a cell incubator for 30 min to solidify the Matrigel. Other operations were the same as the cell migration experiment.

### Sphere Formation Assay

A sphere formation assay was performed, as described (Chen et al., 2018d). In brief, hepatocarcinoma cells were seeded in ultra-low attachment 6-well plates (Corning, #3471) and cultured in Dulbecco’s Modified Eagle’s Medium/F12 (Gibco, #11330032) supplemented with B27 (Thermo Fisher Scientific, #17504044), N2 (Thermo Fisher Scientific, #17502048), 20 ng/ml epidermal growth factor (Thermo Fisher Scientific), and 20 ng/ml basic fibroblast growth factor (Thermo Fisher Scientific). Cells were incubated in a CO_2_ incubator, and 3 days later spheres were counted using an inverted microscope.

### Statistics

Statistical significance was evaluated using a two-tailed Student’s t test. *p* values <0.05 were considered significant.

### Study Approval

Human liver cancer specimens were obtained from the Department of Hepatobiliary Surgery, The First Affiliated Hospital of Zhengzhou University with informed consent, according to the Institutional Review Board approval.

## Results

### Bioinformatics Screening of Hepatocellular Carcinoma hub Genes

**FIGURE 1:**
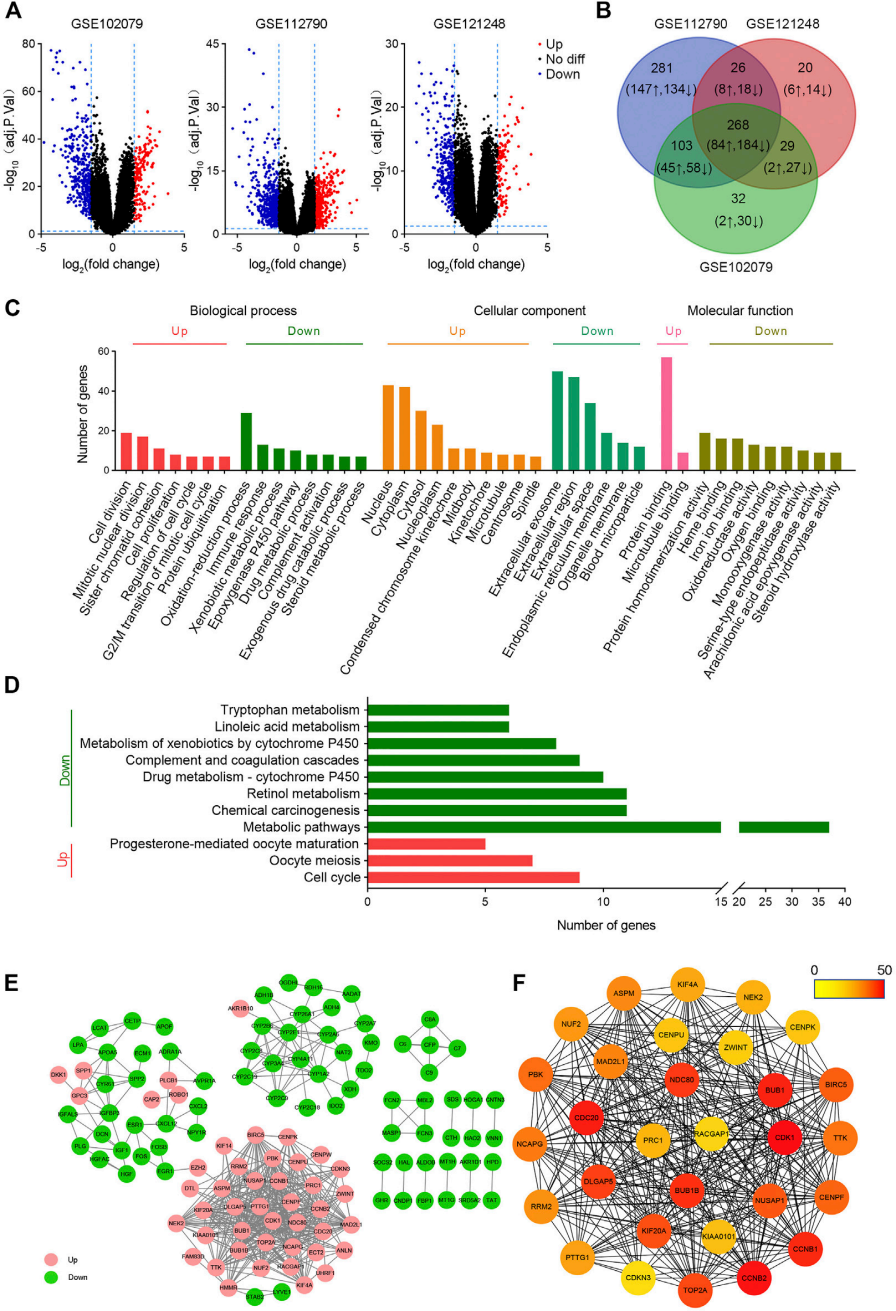
Bioinformatic screening of HCC hub genes. **(A)** Volcanic maps of differentially expressed genes in HCC in GSE102079, GSE112790, and GSE121248 data sets. The *p* value < 0.05 and |logFC| > 1.5 were used as the selection criteria. **(B)** Venn plots of differentially expressed genes in GSE102079, GSE112790, and GSE121248 data sets. **(C)** GO analysis of common differentially expressed genes. **(D)** KEGG analysis of common differentially expressed genes. **(E)** Protein–protein interaction networks of differentially expressed genes. **(F)** Top 30 hub genes were screened by MCC. The color of the circle represents the number of proteins that interacts with it.

To screen DEGs between HCC and non-tumor tissues, three cohorts (GSE102079, GSE112790, and GSE121248) were selected for further analysis. From GSE102079, GSE112790, and GSE121248, 580 DEGs (163 upregulated and 417 downregulated), 948 DEGs (355 upregulated and 593 downregulated), and 462 DEGs (123 upregulated and 339 downregulated) were screened, respectively (Figure 1A). The intersection of DEGs in three data sets was further visualized using the Venny map (https://bioinfogp.cnb.csic.es/tools/venny/index.html). Finally, 268 common DEGs were identified, including 84 upregulated and 184 downregulated genes (Figure 1B).

**FIGURE 2:**
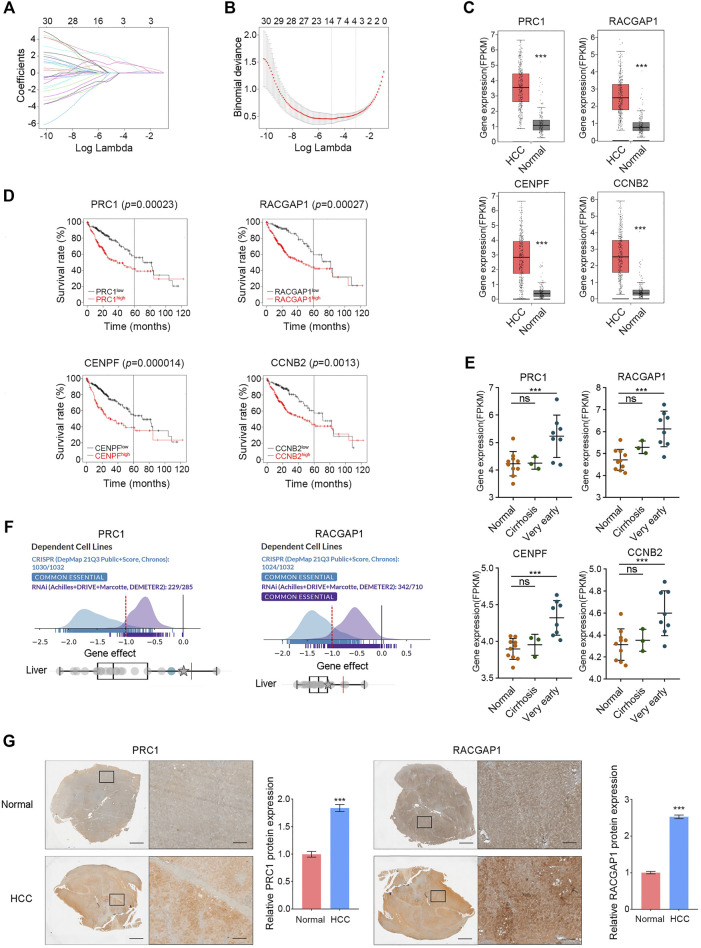
Validation of hub genes in HCC. **(A,B)** PRC1, RACGAP1, CENPF, and CCNB2 were screened by LASSO. **(C)** In the GEPIA database, high expression of PRC1, RACGAP1, CENPF, and CCNB2 in HCC was verified. **(D)** Overexpression of PRC1, RACGAP1, CENPF, and CCNB2 is associated with poor OS in HCC patients. **(E)** Compared with normal tissues, PRC1, RACGAP1, CENPF, and CCNB2 were overexpressed in very early HCC samples, but there was no significant difference in cirrhosis samples. **(F)** PRC1 and RACGAP1 were shown as essential genes in the DepMap database. **(G)** Quantitative immunohistochemical study of PRC1 and RACGAP1 in HCC and normal liver tissues. Scale bars, overall plots, 1 mm, local plots, 100 μm. ns *p* ≥ 0.05; ***p < 0.001, data are shown as mean ± s.d.

GO and KEGG analyses of DEGs were performed on DAVID. As shown in Figure 2C, upregulated BPs mainly include cell division, mitotic nuclear division, and sister chromatid cohesion, while downregulated BPs mainly include the oxidation–reduction process, immune response, and xenobiotic metabolic process; upregulated CCs mainly include nucleus, cytoplasm, cytosol, and nucleoplasm, and downregulated CCs mainly include extracellular exosome, extracellular region, extracellular space, and endoplasmic reticulum membrane; upregulated MFs are protein binding and microtubule binding, and downregulated MFs show the protein homodimerization activity, heme binding, and iron ion binding. In addition, the upregulated DEGs were related to three KEGG pathways, including cell cycle, oocyte meiosis, and progesterone-mediated oocyte maturation, while the downregulated genes were significantly enriched in metabolic pathways, retinol metabolism, and chemical carcinogenesis (Figure 1D).

In order to investigate the function of these genes, a PPI network with 123 nodes and 516 edges was constructed using cytoscape (Figure 1E). These 123 nodes and 516 edges represent 123 proteins and 516 PPI, respectively. Then top 30 hub proteins were filtered out using cytoHubba plug-in of cytoscape (Figure 1F). Interestingly, these hub genes are all upregulated DEGs, indicating their predominant oncogenic role in liver tumorigenesis.

### Validation of hub Genes in Hepatocellular Carcinoma

A total of 30 hub genes were analyzed by LASSO, and four target genes were further screened: PRC1, RACGAP1, CENPF, and CCNB2 (Figures 2A,B). All these four genes were included in the top 30 Hub genes of the validation cohorts (GSE45267, GSE62232, and GSE84402) (Supplementary Figure S1A), and the high expression of these four genes in HCC was also confirmed in the GEPIA database (Figure 2C). Subsequently, a survival analysis of these four hub genes was carried out with the Kaplan–Meier plotter, and the overexpression of them was significantly associated with poor survival in HCC patients (Figure 2D). Part of the occurrence of HCC is associated with further deterioration of liver cirrhosis. We analyzed the expression of these four genes in a normal liver, liver cirrhosis, and very early HCC tissues with data set GSE6764, and the results showed that these four genes were highly expressed in very early HCC tissues but not in liver cirrhosis (Figure 2E). This study revealed that PRC1, RACGAP1, CENPF, and CCNB2 could sensitively distinguish HCCfrom cirrhosis, and they may be potential targets for early HCC detection. In the DepMap database, we analyzed the importance of these four genes in cell survival, and the results showed that PRC1 and RACGAP1 are common essential genes in cell survival, including liver cancer cells (Figure 2F). Subsequently, the high expression of PRC1 and RACGAP1 in HCC was further verified by immunohistochemical results of HCC samples and normal adjacent liver samples (Figure 2G).

### PRC1 and RACGAP1 Drive the Propagation of Liver Cancer

**FIGURE 3:**
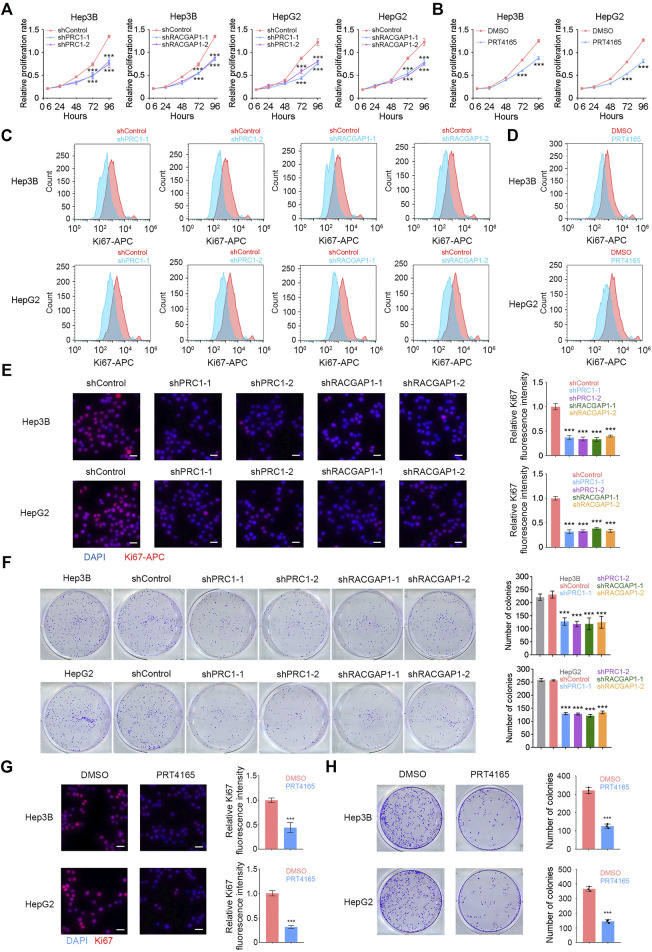
PRC1 and RACGAP1 drive the propagation of liver cancer. **(A,B)** (A) Two thousand Hep3B or HepG2 cells with PRC1 or RACGAP1 knockdown and **(B)** two thousand Hep3B or HepG2 cells treated with 10 μM PRT4165 or DMSO were seeded in 96-well plates and detected by CCK-8 at 6, 24, 48, 72, and 96 h, respectively. The cell proliferation rate was denoted by OD450. **(C,D)** (C) 1 × 10^4^ PRC1 or RAPGAP1 depleted Hep3B (upper panels) and HepG2 (lower panels) cells; **(D)** 1 × 10^4^ Hep3B (upper panels) and HepG2 (lower panels) cells added 10 μM PRT4165 or DMSO were used for intracellular staining for Ki-67. **(E,G)** (E) 1 × 10^5^ PRC1 or RAPGAP1 depleted Hep3B (upper panels) and HepG2 (lower panels) cells; **(G)** 1 × 10^5^ Hep3B (upper panels) and HepG2 (lower panels) cells added 10 μM PRT4165 or DMSO were seeded with 24-well plate slides and used immunofluorescence staining for Ki-67. Scale bars, 30 μm. **(F,H)** (F) Five hundred PRC1 or RAPGAP1 depleted Hep3B (upper panels) and HepG2 (lower panels) cells; **(H)** five hundred Hep3B (upper panels) and HepG2 (lower panels) cells added 10 μM PRT4165 or DMSO were seeded with 24-well plate and stained with crystal violet after 10 days. The number of colonies indicates cell proliferation ability. ***p < 0.001, data are shown as mean ± s.d. Data are representative of at least three independent experiments.

In order to verify the function of PRC1 and RACGAP1 in HCC cells, we designed two shRNAs for each of these two genes, and constructed PRC1 knockdown and RACGAP1 knockdown cells using Hep3B and HepG2 cell lines. Both genes were significantly silenced in the corresponding knockdown cell lines (Supplementary Figures S1B,C), which showed an impaired proliferation capacity (Figure 3A). PRT4165 is an inhibitor of PRC1 (Ismail et al., 2013), and the propagation ability of HCC cells treated by PRT4165 was also significantly reduced (Figure 3B). We labeled the cells with Ki-67 antibody indicating cell proliferation and detected them by flow cytometry and immunofluorescence (Figures 3C,E). It was confirmed that PRC1 and RACGAP1 promoted proliferation. We also performed the clone formation assay and found a decreased clone formation capacity on PRC1, RACGAP1 knockdown, and PRT4165 treatment (Figures 3F,H). Cells treated with PRT4165 showed the same phenotypic changes (Figures 3D,G). These data suggest that PRC1 and RACGAP1 promote the growth and proliferation of liver cancer.

### PRC1 and RACGAP1 Increase the Metastasis of Liver Cancer

**FIGURE 4:**
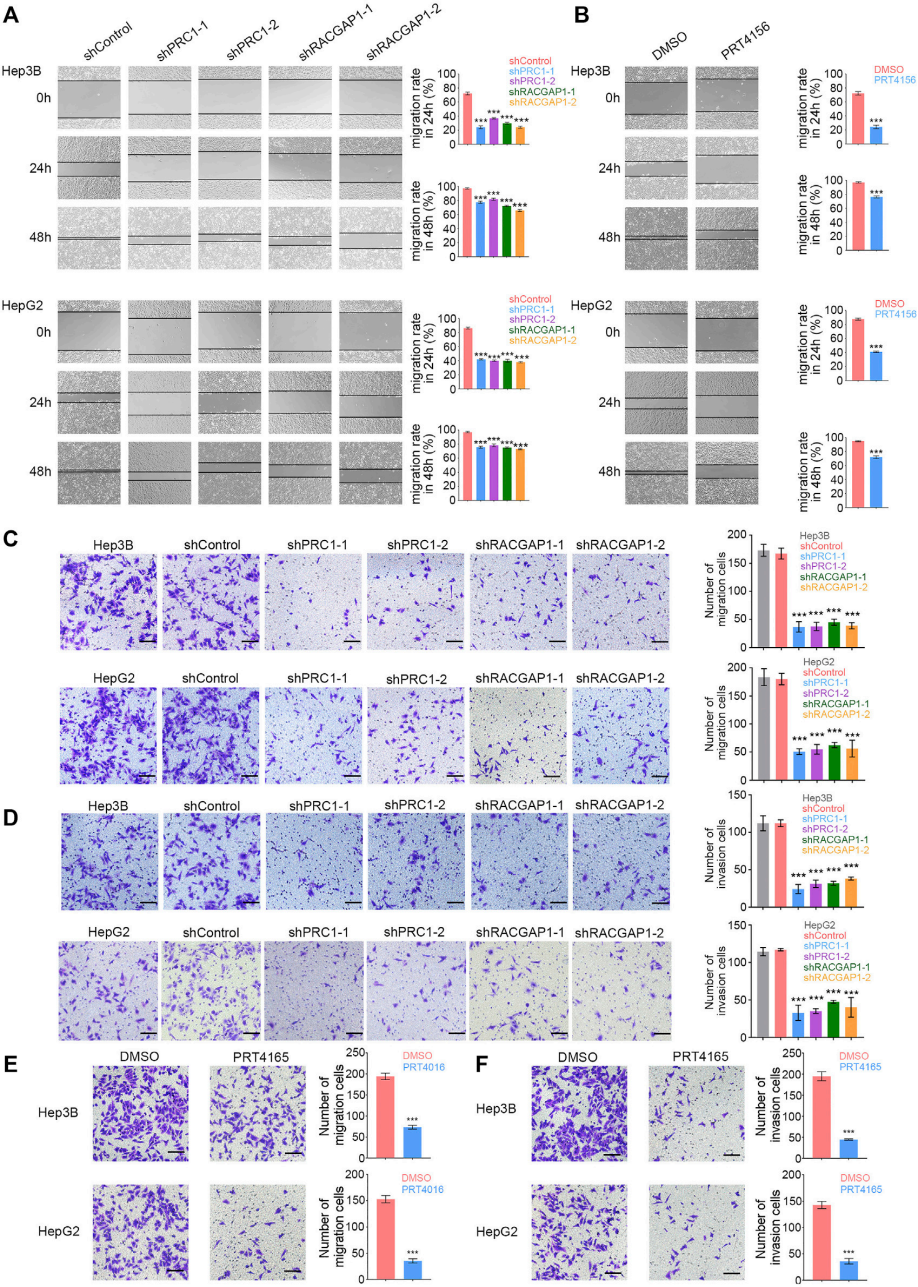
PRC1 and RACGAP1 increase the metastasis of liver cancer. **(A,B)** (A) 1 × 10^5^ PRC1 or RAPGAP1 knockdown Hep3B (upper panels) and HepG2 (lower panels) cells; **(B)** 1 × 10^5^ Hep3B (upper panels) and HepG2 (lower panels) cells treated with 10 μM PRT4165 or DMSO were seeded in 24-well plates. The cells were scratched with 10 μL tips and photographed at 0 and 24 h. The migration rate was obtained by the formula: (Width0h−Width24h)/Width0h. **(C,E)** (C) 2 × 10^4^ PRC1 or RAPGAP1 knockdown Hep3B (upper panels) and HepG2 (lower panels) cells; **(E)** 2 × 10^4^ Hep3B (upper panels) and HepG2 (lower panels) cells treated with 10 μM PRT4165 or DMSO were seeded to the upper chamber and stained with crystal violet after 24 h. **(D,F)** (D) 2 × 10^4^ PRC1 or RAPGAP1 knockdown Hep3B (upper panels) and HepG2 (lower panels) cells; **(F)** 2 × 10^4^ Hep3B (upper panels) and HepG2 (lower panels) cells treated with 10 μM PRT4165 or DMSO were seeded to the upper chamber treated with Matrigel and stained with crystal violet after 24 h. Scale bars, 100 μm. ***p < 0.001, data are shown as mean ± s.d. Data are representative of at least three independent experiments.

We also detected the role of PRC1 and RACGAP1 in tumor metastasis. The wound-healing assay revealed that PRC1, RACGAP1 silencing, and PRT4165 treatment significantly decreased the cell metastasis rate (Figures 4A,B). We also found that PRC1 or RACGAP1 depletion dramatically reduced the migration and invasion of Hep3B and HepG2 cells with Transwell assays (Figures 4C–F). Therefore, these data suggest that PRC1 and RACGAP1 increased metastasis of liver cancer.

### PRC1 Promotes the Self-Renewal of Liver CSCs Through Wnt Signaling

**FIGURE 5:**
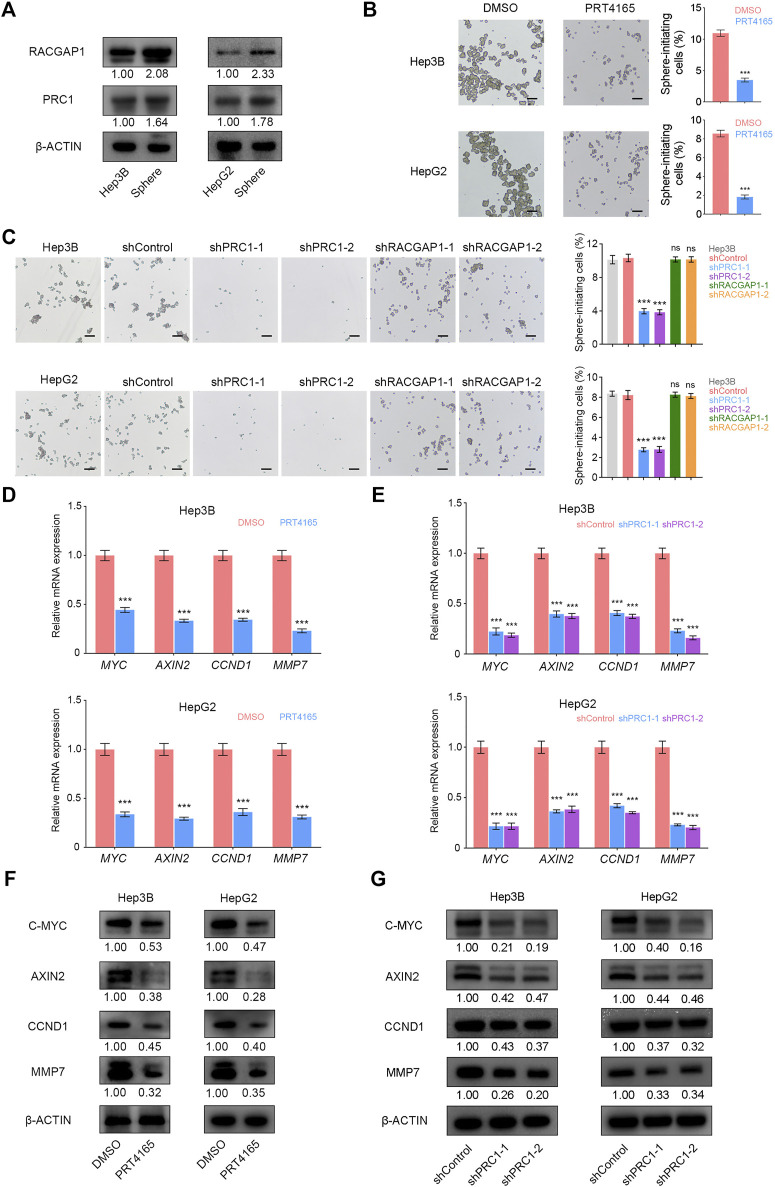
PRC1 promotes the self-renewal of liver CSCs through Wnt signaling. **(A)** High expression of PRC1 and RACGAP1 in liver CSCs was detected by Western blot. **(B,C)** (B) 2 × 10^4^ PRC1 or RAPGAP1 knockdown Hep3B (upper panels) and HepG2 (lower panels) cells; **(C)** 2 × 10^4^ Hep3B (upper panels) and HepG2 (lower panels) cells treated with 10 μM PRT4165 or DMSO were seeded in ultra-low attachment 6-well plates and cultured in Dulbecco’s Modified Eagle’s Medium/F12 supplemented with B27, N2, 20 ng/ml epidermal growth factor, and 20 ng/ml basic fibroblast growth factor. After three days, the spheres were examined under a microscope. **(D,E)** qRT-PCR was used to verify the mRNA expression of *AXIN2*, *MYC*, *CCND1*, and *MMP7*. **(F,G)** Protein level expression of AXIN2, C-MYC, CCND1, and MMP7 was verified by Western blot. Scale bars: 100 μm. ns *p* ≥ 0.05; ***p < 0.001, data are shown as mean ± s.d. Data are representative of at least three independent experiments.

PRC1 and RACGAP1 are highly expressed in liver CSCs (Figure 5A), so we then detected whether PRC1 and RACGAP1 play a critical role in the stemness of Hep3B and HepG2 cells using the sphere formation assay. The results showed that PRC1 knockdown or PRT4165 treatment significantly reduced the sphere formation capacity of Hep3B and HepG2 cells, while RACGAP1 did not (Figures 5B,C). Therefore, PRC1 can enhance the stemness of Hep3B and HepG2 cells.

It has been reported that PRC1 promotes the self-renewal ability of lung CSCs through the WNT signaling pathway (Zhan et al., 2017). We detected mRNA and protein expression levels of WNT target genes AXIN2, MYC, CCND1, and MMP7 and found that they were significantly reduced in liver CSCs with PRC1 knockdown and PRT4165 treatment (Figures 5D–G). It is revealed that PRC1 promotes the self-renewal of liver CSCs through the Wnt signaling pathway.

## Discussion

**FIGURE 6:**
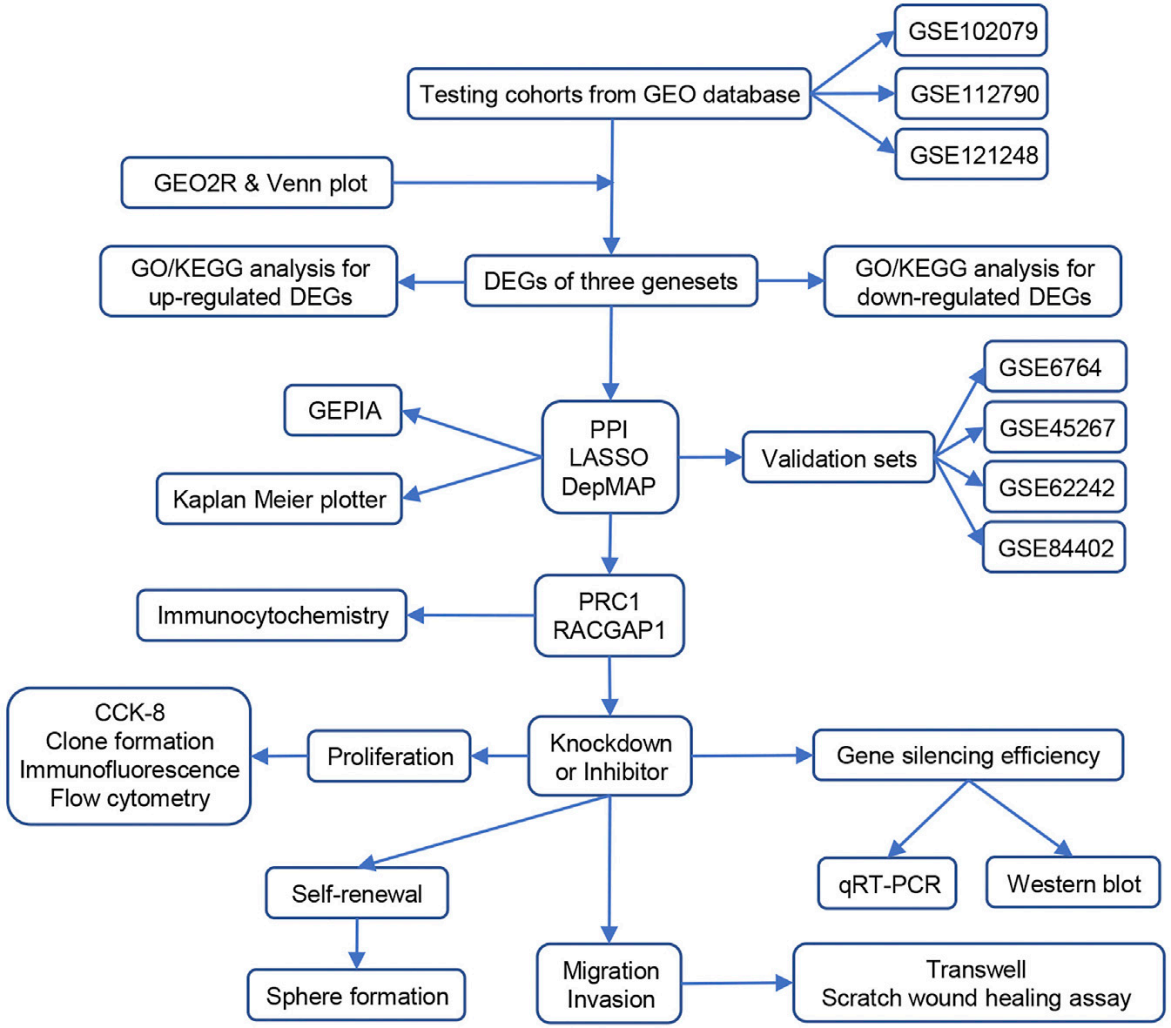
Schematic illustration of data processing and experimental verification of this study.

As a predominant type of liver cancer, HCC has a very high cancer mortality rate, which is mainly due to tumor heterogeneity and lack of early diagnosis. HCC patients are generally in an advanced HCC stage when they are detected and thus miss the best time of therapy (Llovet et al., 2018). In addition, some HCC patients have tumor recurrence or metastasis resulting in death due to lack of appropriate prognostic detection methods after treatment (Wang et al., 2017). Therefore, it is important to find more accurate and reliable biomarkers for the diagnosis and prognosis of HCC. In this study, we selected three HCC data sets from the GEO database and used the GEO2R online analysis to obtain the differential genes between HCC and non-cancer samples. Next, we constructed a PPI network and screened the top 30 hub genes from 268 DEGs. These 30 genes were enriched by LASSO regression and analyzed in the DepMap database, and PRC1 and RACGAP1 were finally screened out as potential targets for early HCC detection. Subsequently, the high expression of PRC1 and RACGAP1 in HCC was verified by immunohistochemistry. We constructed HCC cell lines with PRC1 or RACGAP1 knockdown and performed cell proliferation, metastasis, and stemness experiments, which revealed that PRC1 and RACGAP1 enhanced the propagation and metastasis of HCC. In addition, PRC1 promoted the self-renewal ability of liver CSCs through the WNT signaling pathway (Figure 6).

Taking advantage of the bioinformatic analysis and functional validation, we have identified PRC1 and RACGAP1 as diagnosis markers and therapeutic targets in this study. The polycomb repressive system plays a fundamental role in controlling gene expression during mammalian development (Blackledge et al., 2020). To achieve this, polycomb repressive complexes 1 and 2 (PRC1 and PRC2) bind target genes and use histone modification-dependent feedback mechanisms to form polycomb chromatin domains and repress transcription (Fursova et al., 2019). PRC1 is an E3 ubiquitin ligase that mono-ubiquitylates histone H2A at lysine 119 (H2AK119ub1), which is regulator of gene expression required for embryonic stem cell (ESC) fate decisions during development (Tamburri et al., 2020). PRC1 also affects the function of certain types of adult stem cells, and their misregulation contributes to tumorigenesis in several tissues (Jia et al., 2021). Rac GTPase activation protein 1 (RACGAP1) is a component of the centralspindlin complex that serves as a microtubule-dependent and Rho-mediated signaling required for the myosin contractile ring formation during the cell cycle cytokinesis (Wang et al., 2019). RACGAP1 was initially identified in testis and male germ cells and has been demonstrated to be a key regulator in various malignancies, such as colorectal cancer (Zhou et al., 2018), ovarian cancer (Wang et al., 2018), meningioma (Ke et al., 2013), uterine cancer (Ebinger et al., 2016), and breast cancer (Ren et al., 2021). In addition, RACGAP1 plays a critical role in mitochondrial fission and speeds up mitochondrial renewal and mitochondrial respiration, which leads to cancer cell metastasis (Zhou et al., 2021).

In past studies, some diagnostic biomarkers for HCC have been found using bioinformatic analyses and experimental verifications. But they are difficult to be transformed into clinical diagnosis and treatment due to their inability to distinguish liver lesions from cancer (De Stefano et al., 2018). According to our experimental results, PRC1 and RACGAP1 are highly expressed in early HCC, while there is no significant difference between normal liver tissues and cirrhotic tissues, which suggests that PRC1 and RACGAP1 have high sensitivity and reliability as biomarkers for early HCC diagnosis. After surgical removal of the tumor, there is still about a 70% chance of recurrence and metastasis, resulting in death of HCC patients (Xu et al., 2019). Our results indicate that PRC1 and RACGAP1 can promote the proliferation and metastasis of HCC. It provides potential targets for controlling the progression of HCC and inhibiting metastasis, which is of great significance for the treatment of HCC and improving the prognosis of patients.

In CSC theory, tumor growth is caused by the continuous proliferation of a class of stem cells, CSCs, similar to normal tissue renewal (Shimokawa et al., 2017; Toh et al., 2017; Chen et al., 2021). It suggests that HCC treatment should not only reduce tumor size but also inhibit the growth of liver CSCs. We found that PRC1 promoted the self-renewal ability of liver CSCs, which is exciting for inhibiting the growth of liver CSCs and improving the therapeutic effect of HCC. Our results also confirmed that PRC1 increases the self-renewal ability of liver CSCs through the WNT signaling pathway, closely related to stemness, which further proved that PRC1 is a reliable therapeutic target for HCC.

In conclusion, PRC1 and RACGAP1 screened by the bioinformatic analysis and experimental verification can promote the proliferation and metastasis of HCC, and PRC1 also improves the self-renewal ability of liver CSCs. Therefore, PRC1 and RACGAP1 are identified as prognosis markers for early HCC and therapeutic targets for liver cancer and liver CSCs.

## Data Availability

The original contributions presented in the study are included in the article/Supplementary Material, further inquiries can be directed to the corresponding authors.

## Supplementary Material

Supplementary Figure S1
**(A)** PRC1, RACGAP1, CENPF, and CCNB2 were included in the TOP 30 hub genes of the validation group (GSE45267, GSE62232, and GSE84402). The color of the circle represents the number of proteins that interact with it. **(B,C)** Knockdown efficiency of PRC1 and RACGAP1 in Hep3B and HepG2 cells. In **(B)** detected by qRT-PCR and in **(C)** detected by Western blot. *** *p* < 0.001, data are shown as mean ± s.d.
