# Supplementary figures and images for: PRC1 and RACGAP1 are Diagnostic Biomarkers of Early HCC and PRC1 Drives Self-Renewal of Liver Cancer Stem Cells
Source: Front Cell Dev Biol. 2022 Apr 4;10:864051. doi: 10.3389/fcell.2022.864051 (PMC9014962; doi:10.3389/fcell.2022.864051)

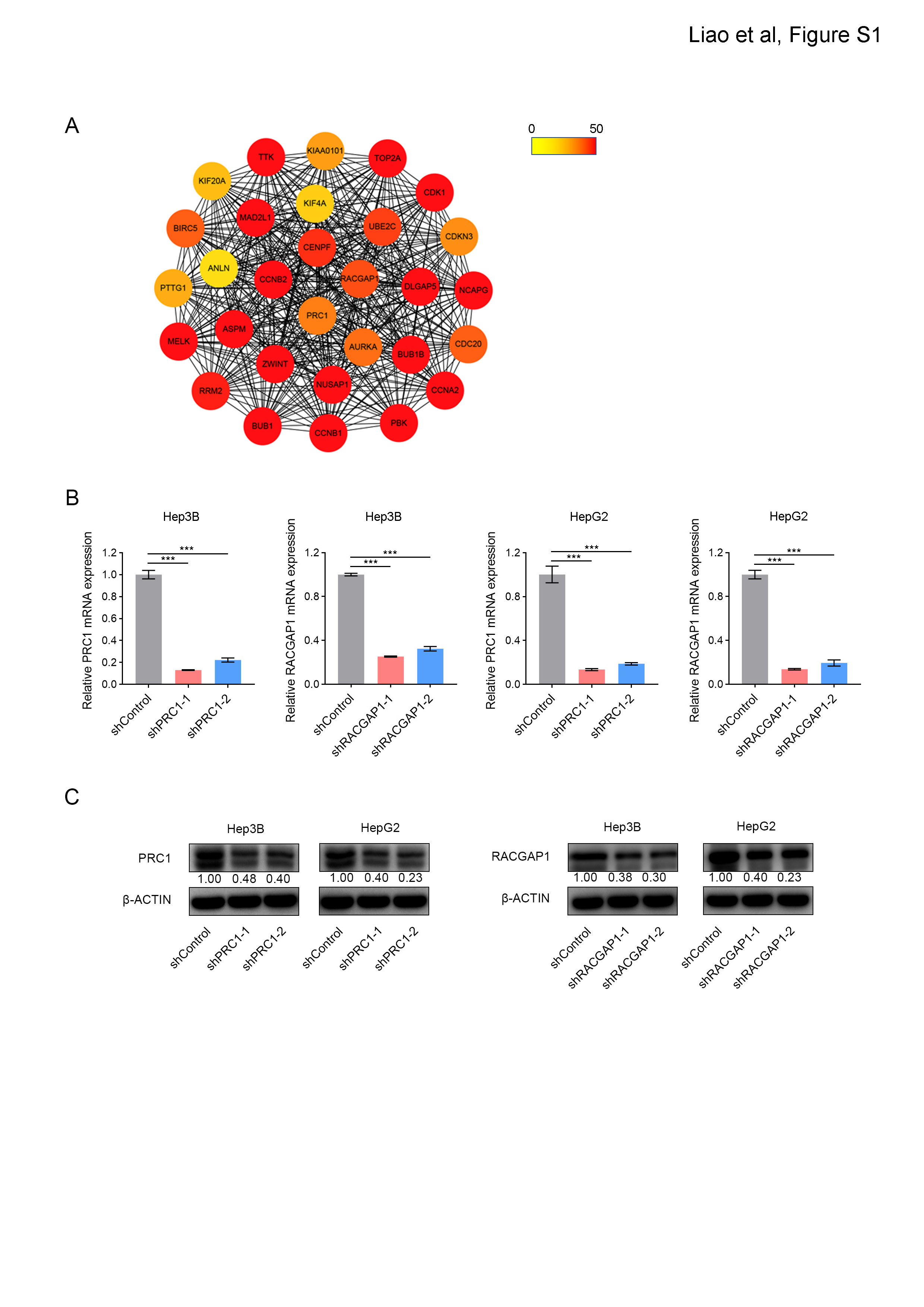

Supplement: Supplementary file 1 [file Image1.JPEG]
